# Embodied-Cognitive Linguistics: Integrating Marxist perspectives on contemporary Cognitive Linguistics theory

**DOI:** 10.3389/fpsyg.2024.1475196

**Published:** 2024-11-01

**Authors:** Honglin Zhou, Xiaoyang Luo

**Affiliations:** School of English Studies, Sichuan International Studies University, Chongqing, China

**Keywords:** Cognitive Linguistics, embodied cognition, Marxist philosophy, linguistic revolution, socio-economic factor

## 1 Three linguistic revolutions in the 20th century

The 20th century witnessed three significant revolutions in the field of linguistics, each reshaping our understanding of language from different theoretical angles: the Structuralist revolution led by Ferdinand de Saussure, the Transformational-Generative revolution pioneered by Noam Chomsky, and the Cognitive Linguistics revolution, which emerged from a philosophical shift toward embodiment (Wang, 2015). Saussure's (1916) structuralist approach marked a departure from historical and comparative linguistics, emphasizing the study of language as a self-contained system, isolated from its social or cognitive contexts. Saussure's Structuralism laid the groundwork for modern linguistics by focusing on language as a system of signs, but it largely excluded human cognition and social contexts.

By the mid-20th century, Chomsky (1957, 1965) revolutionized linguistics again with his Transformational-Generative Grammar, shifting the focus from structural relationships to the innate cognitive mechanisms underlying language. Chomsky proposed that humans are born with a Universal Grammar—a biologically endowed set of linguistic principles that allows them to generate infinite grammatical structures. This theory emphasized competence—the internal knowledge of language—over performance, which refers to actual language use. Chomsky's ideas, grounded in formalism, aligned with the emerging fields of cognitive psychology and artificial intelligence, where the mind was increasingly understood as an information-processing system. His focus on syntax and formal rules, however, largely abstracted language from its socio-cultural context, treating linguistic competence as an autonomous cognitive module, independent of sensory experience and human interaction with the world.

The Transformational-Generative (TG) approach, primarily developed by Chomsky, has been challenged by philosophers like Andy Clark. Clark (1997), a leading figure in the philosophy of mind and cognitive science, argues against the TG view of language, particularly its focus on an abstract, innate grammar. Andy Clark's critique of the Transformational-Generative approach highlights the importance of embodied cognition, arguing that language emerges from the dynamic interplay between mind, body, and environment. While this perspective has helped move away from viewing language as an isolated cognitive module, it still falls short of addressing the broader socio-material conditions that influence linguistic practices.

Cognitive Linguistics, which emerged in the 1980s through the work of scholars like Lakoff and Johnson (1980, 1999), also challenged Chomsky's formalist and nativist perspectives. Cognitive Linguistics views language as shaped by human interaction and perception but often overlooks the influence of socio-material conditions. The cognitive approach contends that meaning is grounded in embodied experience—the sensory and motor activities that enable humans to engage with their environment. Rather than focusing on abstract, universal rules, Cognitive Linguistics highlights the role of metaphor, conceptualization, and embodied cognition in shaping language. For instance, Lakoff and Johnson's (1980) theory of conceptual metaphor posits that many abstract ideas are understood through physical experiences, such as how we conceptualize time as a linear progression (e.g., “time flies”) based on our embodied experience of motion. Cognitive Linguistics thus emphasizes performance—the actual use of language in real-world contexts—arguing that language is learned, processed, and used dynamically in social and cultural interactions. This shift in perspective positioned Cognitive Linguistics as an interdisciplinary bridge between linguistics, psychology, and philosophy, moving beyond the narrow focus on syntax that characterized Chomskyan linguistics. By focusing on the psychological reality of language, Cognitive Linguistics provided a more holistic understanding of how language emerges from the interaction between the mind, body, and environment. Unlike Chomsky's formalist model, which considers language to be a cognitive phenomenon isolated from experience, Cognitive Linguistics suggests that linguistic structures and meanings are deeply rooted in human embodiment, shaped by interactions with the physical, social, and cultural world.

To summarize, the Structural Linguistics merely investigated the “language” element, excluding the factor of human beings. TG Linguistics added “human mind” into linguistic research. However, they still admitted the innateness and autonomy of mind and language. Cognitive Linguistics and Embodied-Cognitive Linguistics hold an opposing opinion that they highlight the embodiment of language, thus introducing “reality” into linguistic study. The central aim of this paper is to argue that integrating Marxist dialectical materialism into Cognitive Linguistics, particularly as informed by the theory of embodied cognition, provides a more comprehensive framework for understanding language. While Cognitive Linguistics successfully ties meaning to embodied experience, it overlooks how language is shaped by socio-economic conditions and power dynamics. By bringing in Marxist theory, the Embodied-Cognitive Linguistics address these gaps and provide a more holistic account of language as a cognitive capacity, deeply embedded in both material and social realities.

## 2 Cognitive Linguistics and its challenges

Evans (2012) identified two key commitments in Cognitive Linguistics: the Cognitive Commitment and the Generalization Commitment. The Cognitive Commitment ensures that linguistic theories are consistent with our understanding of human cognition, framing language as part of general cognitive processes like perception and memory. Conversely, the Generalization Commitment seeks to unify principles across all linguistic areas, including syntax, semantics, and phonology, rather than treating them as separate systems. These commitments ground Cognitive Linguistics within broader cognitive science and emphasize its holistic approach to language understanding. Recently, Pelkey (2023) conducted a historical survey on embodied cognition and language, concluding that real-world experiences can reconnect the body and mind. Imaginative and rational thoughts are processed within the same frameworks of movement and memory, suggesting that even conventional form-content relationships in language can be viewed as networks of individual or interpersonal experiences.

One of the key challenges faced by contemporary linguistic theory is its neglect of the social and material conditions under which language develops and is used. Cognitive Linguistics, with its emphasis on the embodiment of language, has provided groundbreaking insights into how language arises from human cognitive processes. Scholars like Lakoff and Johnson (1980, 1999) have demonstrated that language is not an abstract, autonomous system; rather, it is deeply intertwined with bodily experiences and sensory perceptions. However, the field is often criticized for underestimating social and material dimensions, focusing too much on individual cognition. While embodied cognition acknowledges the role of sensory experience in shaping thought, it often portrays language acquisition and use as primarily cognitive activities occurring within isolated individuals. This perspective neglects the crucial social contexts in which language operates. As Dabrowska (2016) argues, focusing on internal cognitive processes risks overlooking how language is learned and used through social interaction and cultural practices. Additionally, Cognitive Linguistics has been criticized for neglecting socio-economic and material factors, failing to account for how these conditions shape linguistic meaning (Lecercle, 2006). A further criticism involves the underestimation of power dynamics in language use, as Cognitive Linguistics often treats language as a neutral reflection of cognitive processes rather than recognizing the influence of social power relations.

## 3 The amendment of Embodied-Cognitive Linguistics

Embodied cognition is a central theory within Cognitive Linguistics. Since its introduction by Lakoff and Johnson (1999), numerous studies have sought to deepen its understanding. Gibbs (2003) first highlighted the role of embodied experience in comprehending linguistic meaning, arguing against earlier cognitive theories that posited meanings as purely propositional and abstract. He demonstrated, through various linguistic and psychological experiments, that embodied perception is crucial for understanding linguistic elements, ranging from words to texts, suggesting that concepts are fundamentally grounded in bodily experiences. Later, Barsalou (2008) systematically reviewed grounded cognition theory, which posits that concepts and thoughts are bodily simulated. This means that the brain processes experiences as multimodal representations stored in memory. When relevant knowledge is needed, these representations are reactivated, allowing the brain to simulate the original experience. Lakoff (2012) expanded on this with a neural theory of thought and language (NTTL), asserting that thought is physical and realized through neural circuitry linked to bodily experiences. This processing applies to both abstract concepts and language.

Wang (2019) proposed a new framework called “Embodied-Cognitive Linguistics,” aiming to address the shortcomings of contemporary Cognitive Linguistics by incorporating elements of Marxist dialectical materialism and social practice theory. This innovative approach emphasizes that language is not merely a cognitive phenomenon but also a product of human social interactions and economic conditions (Marx, 1867; Wang, 2021). While traditional theories often reduce the objective dimensions of language to interactions among individuals, the Embodied-Cognitive Linguistics framework extends the cognitive approach by establishing a dialectical relationship between subjective cognitive experiences and objective social-material realities. This approach recognizes that language is not only shaped by embodied cognitive processes but also by the material conditions under which people live. Marxist historical materialism posits that human ideas, including language, are shaped by material conditions and social structures (Marx and Engels, 1846). This conception of “humanized nature” suggests that language development is a process influenced by human interaction with the environment, shaped not only by cognitive experiences but also by social and economic conditions. This perspective recognizes that language reflects and reinforces the power dynamics inherent in society, connecting meaning not only to individual cognition but also to the material conditions and social structures that influence language use. Integrating Marxist elements, Embodied-Cognitive Linguistics offers a more comprehensive view of how language arises from embodied cognition and social-material factors. Empirical studies have strongly evidenced this opinion (Chow et al., 2014; Giacobbe et al., 2022; Knoeferle et al., 2022; Serafini, 2017). Besides, cognitive methods such as “ontological metaphor” are used to explain abstract concepts through “fetishism.” Abstract, invisible, and unfamiliar concepts in language are mostly comprehended through the use of metaphor and metonymy mechanisms, with the help of concrete, visible and familiar items. The physical and mental status of human beings cannot be separated from space and concrete materials, and cannot be separated from metaphors. If there were no metaphors, there would be no abstract thinking (Lakoff and Johnson, 1999).

Postmodernist philosophers reject both monism and dualism, advocating instead for pluralism. This perspective, known as “perspectivism,” suggests that when people observe the same phenomenon from different viewpoints, they inevitably have varied feelings and insights shaped by their unique positions. This idea challenges the notions of metaphysics and absolute truth, emphasizing the importance of subjective interpretations. Embodied-Cognitive Linguistics aligns with postmodern philosophical concepts such as anti-foundationalism, decentralization, and pluralism. It posits that there is no singular, unified essence in the world, nor is there a necessity to adhere to a central truth. Drawing from postmodernist thought, Embodied-Cognitive Linguistics introduces the metaphorical “View of Elephant and Leopard” (Wang, 2021; Qian, 2022). This concept is illustrated by two well-known Chinese idioms: “The blind touch the elephant” and “Seeing a leopard by peeking at a spot.” Both idioms critique the tendency to overgeneralize based on limited perspectives. Embodied-Cognitive Linguistics embraces a postmodern view, recognizing that individuals cannot grasp everything simultaneously; rather, understanding often requires piecing together insights gained from specific parts to form a more complete picture.

Embodied cognition theory has successfully addressed the limitations of objectivism and formalism in traditional linguistic research, ushering in a new direction for the field. Within this framework, Embodied-Cognitive Linguistics aims to amend contemporary Cognitive Linguistics by elucidating the intricate relationships between “reality,” “cognition,” and “language.” Importantly, it integrates fresh perspectives from Marxism and perspectivism, suggesting that understanding language as a cognitive capacity can benefit from a Marxist lens, which emphasizes the social and material contexts that shape linguistic meaning. This article shows that Marxism complements Cognitive Linguistics, enhancing its explanatory power and addressing unresolved issues. By incorporating social-material factors, Embodied-Cognitive Linguistics accounts for how language is influenced by both embodied cognition and the material conditions shaping human life. This dual approach provides a more holistic understanding of language, recognizing that linguistic practices are shaped by cognitive processes, socio-economic influences, and Power dynamics.

While the theory is still developing, it highlights a significant trend in future research. Furthermore, engaging with potential objections—such as the criticisms regarding the neglect of linguistic structure or the question of why humans possess unique language-learning capabilities compared to other animals—will strengthen this framework. By incorporating these discussions, the paper aims to provide a more robust contribution to the ongoing discourse in linguistics (Niu, 2021).
